# Building Wellbeing in People With Chronic Conditions: A Qualitative Evaluation of an 8-Week Positive Psychotherapy Intervention for People Living With an Acquired Brain Injury

**DOI:** 10.3389/fpsyg.2020.00066

**Published:** 2020-01-31

**Authors:** Chloe Tulip, Zoe Fisher, Helen Bankhead, Lowri Wilkie, Julia Pridmore, Fergus Gracey, Jeremy Tree, Andrew H. Kemp

**Affiliations:** ^1^Department of Psychology, College of Human and Health Sciences, Swansea University, Swansea, United Kingdom; ^2^Health and Wellbeing Academy, College of Human and Health Sciences, Swansea University, Swansea, United Kingdom; ^3^Community Brain Injury Service, Swansea Bay University Health Board, Morriston Hospital, Swansea, United Kingdom; ^4^School of Psychology, Cardiff University, Cardiff, United Kingdom; ^5^Norwich Medical School, University of East Anglia, Norwich, United Kingdom

**Keywords:** acquired brain injury, ABI, chronic conditions, positive psychotherapy, PPT, healthcare improvement

## Abstract

Research indicates that Acquired Brain Injury (ABI) is associated with significant and chronic impairment across multiple areas of functioning including physical, cognitive, emotional and behavioral domains. Whilst impairments associated with ABI can be ameliorated, cure is seldom possible. The emergence of positive psychology reflects a paradigm shift in health and wellbeing research, highlighting the role of character strengths, positive emotions, meaning, and resilience. Positive psychology interventions have been demonstrated to improve wellbeing in a variety of populations, although research investigating the impact of positive psychotherapy for people living with ABI are sparse. Here we characterize the experience of an 8-week positive psychotherapy intervention for 13 people living with ABI including four mentors and nine participants using thematic analysis of transcripts collected during mini-groups and one-to-one interviews. Six main themes were identified including empowerment, social opportunity, coping, cultivation of positive emotion, consolidation of skills and barriers. Results indicated that wellbeing can be promoted and improved in individuals with ABI. Recent theoretical developments in wellbeing science highlight scope to improve the intervention by connecting individuals to their communities and spending time in nature.

## Introduction

Acquired Brain injury (ABI) refers to injury to the brain that occurs after birth and can be subdivided Traumatic Brain Injury (TBI) or Non-traumatic Brain Injury (NTBI; Saatman et al., 2008). TBIs occur when an external force injures the brain. NTBIs occur both from an internal or external source, but are not directly caused by external force, (e.g., stroke, hypoxia, encephalitis, etc.). ABI can be associated with significant and chronic impairments across multiple areas of functioning including physical, cognitive, emotional, behavioral and social domains (Milders et al., 2003; Barman et al., 2016; de Freitas Cardoso et al., 2019; Kane et al., 2019). Critically, the psychological consequences of ABI are generally hidden and are associated with poor involvement in rehabilitation, hospital re-admission, long-term disability, limited social activity, reduced ability to manage physical conditions, increased health service usage, suicide and a general increase in risk for mortality (Gillen et al., 2001; Naylor et al., 2012; Ayerbe et al., 2013; van Eeden et al., 2016). People affected by ABI – as with other chronic conditions – have little access to psycho-social interventions to address ongoing holistic needs: almost three- quarters of people living with ABI feel that their psychological needs are not met (McKevitt et al., 2011; Oyesanya, 2017). The main goal of the present study therefore is to report a qualitative evaluation of routinely collected data on participant experience of an 8-week positive psychotherapy intervention.

Healthcare systems are typically founded on the acute medical model, which tackle illnesses and conditions by adopting a “find it and fix it” approach (Keller and Carroll, 1994). Although a disease focus is advantageous when delivering life-saving care and attempting medical stability in the acute stage, this model does not adequately support the holistic needs of individuals during the post-acute and community-based stages. For example, disease focused approaches aim to reduce impairment or distress associated with a disease but do not seek to build wellbeing, facilitate psychological adjustment and community re-integration. With respect to ABI, neurorehabilitation services typically adopt a disease focus with an emphasis on reducing impairment through compensatory or restorative techniques. The dominant model used to treat psychological difficulties post ABI has been Cognitive Behavioral Therapy (CBT). CBT aims to alter unhelpful negative thoughts in order to reduce negative affect and psychological distress. These disease focused approaches are underpinned by the assumption that eradication or reduction of impairment will improve health and wellbeing. However, this is problematic for two reasons; firstly, chronic conditions cannot be “fixed” and secondly, the absence of impairment does not equate to wellbeing (Anderson, 1995). Accordingly, there is a need for services to focus on reducing impairment, while also focusing on creating a context for acceptance and wellbeing despite impairment (Schretlen and Shapiro, 2003; Dikmen et al., 2009).

It is now widely accepted that health and wellbeing are no longer tied to the presence or absence of illness or disease (Anderson, 1995). The World Health Organisation (WHO) defined health as “a state of complete physical, mental and social wellbeing and not merely the absence of disease or infirmity” (World Health Organization [WHO], 1948). Whilst this definition goes beyond defining health as an absence of disease, as per the medical model, there are several issues. Striving for “complete” health as a goal for health pathologizes suboptimal health states, thereby contributing to the over-medicalization of society (Hanslik and Flahault, 2016). In addition, the word “complete” is arbitrary, it is difficult to ascertain a “complete” sense of wellbeing and implies that most of us are unhealthy, most of the time. This approach to health and wellbeing is particularly problematic for people with chronic conditions who experience issues in physical, mental and social domains. Under this definition, wellbeing is not attainable for people with chronic conditions. Fortunately, research has highlighted the shortcomings of the WHO definition of health and wellbeing, giving rise to alternate theories and subsequent definitions.

Here we highlight two approaches to the study of wellbeing: hedonic and eudaimonic theories. Hedonic theories of wellbeing, such as Subjective Wellbeing theory (SWB; Diener, 1984), focus on three main components: a cognitive aspect involving global appraisals of life satisfaction and two emotional components, including positive affect and negative affect (Diener, 1984). Another well described hedonic theory of emotion is Barbara Fredrickson’s *Broaden and Build Model* (2001), which highlights a role for positive emotions in broadening our thought-action repertoire leading to more creative and flexible actions. This model further emphasizes the role of positive emotions in building cognitive, psychological, social and personal resources that promote wellbeing and psychological resiliency. Research has identified links between positive emotion, positive health outcomes and increased social connectedness (DuBois et al., 2012). Positive psychological attributes – including optimism and curiosity – have also linked to a decreased risk for disease development and increased engagement in positive health behaviors (Boehm and Kubzansky, 2012; Richman et al., 2005). By contrast, eudaimonic theories, such as Psychological Wellbeing theory (PWB; Ryff, 1995), emphasise purpose and meaning in life. Intriguingly, recent epidemiological research on the United States Health and Retirement Study has identified associations between stronger life purpose and lower mortality (Alimujiang et al., 2019), highlighting an intimate connection between mental wellbeing and physical health. Ryff (1995) proposed six components of PWB which include; self-acceptance, positive relationships with others, autonomy, personal growth, environmental mastery and purpose in life. In 2011, Martin Seligman introduced the *PERMA model*, which combines hedonic and eudaimonic aspects of wellbeing, presenting a model of wellbeing that is comprised of positive emotions, engagement, relationships, meaning and accomplishments. These five core elements of wellbeing according to PERMA theory are not mutually exclusive, and Seligman has argued that flourishing arises when a person excels across all five of these pillars.

**FIGURE 1:**
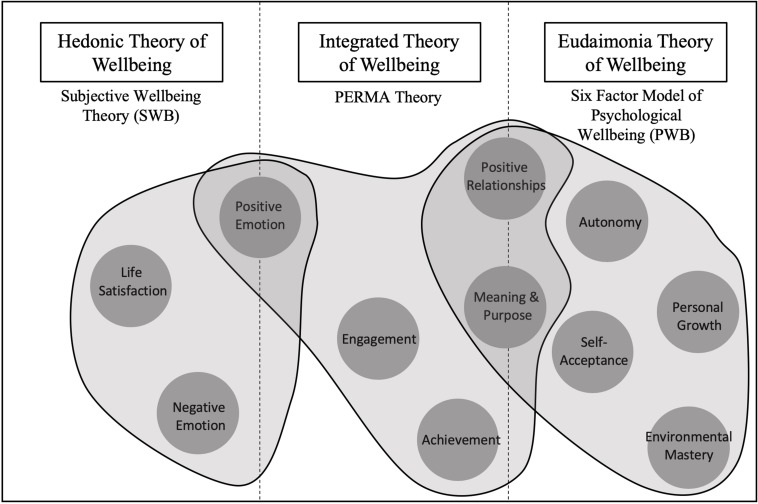
Factors of Wellbeing According to Hedeonic (Subjective Wellbeing Theory), Eudaimonic (Six factor model of Psychological Wellbeing) and Integrated Approaches (PERMA Theory).

All these models of wellbeing present evidence-based factors that contribute to wellbeing (Figure 1). A commonality between these three models is that they all adopt principles relating to Positive Psychology (PP). Positive Psychology is a unique discipline that focuses on identifying human strengths and factors that contribute to a life well-lived (Seligman, 2011). A key principle of PP is that strengths and virtues can be mobilized to develop wellbeing. For example, strategies include practicing optimistic thinking and mindfulness (James, 2011), savoring positive events through diary writing (Sherliker and Steptoe, 2000; Harris et al., 2003; Ben-Shahar, 2010; Jose et al., 2012), making a gratitude visit, (Emmons and Crumpler, 2000; Sansone and Sansone, 2010; Emmons and Stern, 2013), discovering and employing character strengths, (Tedmanson and Guerin, 2011; Panter-Brick and Leckman, 2013), and using relaxation techniques such as guided imagery (Larsson and Starrin, 1992; Gedde-Dahl and Fors, 2012). Published meta-analyses of techniques report enhanced wellbeing and decreased depressive symptoms (Sin and Lyubomirsky, 2009; Bolier et al., 2013; Chakhssi et al., 2018).

The emergence of positive psychology, and related bodies of literature including Quality of Life (QoL), patient centerd care and patient activation reflect a paradigm shift in health and wellbeing research. There is now a focus on creating a context for wellbeing that spans beyond attempts to simply eradicate or reduce impairment. For example, developments in QoL literature for those living with ABI emphasize the need for more comprehensive assessments that include social inclusion, community integration, human rights and personal development (Fernández et al., 2019). This psychosocial approach to assessment reaches beyond disease-specific assessments (Sánchez, 2010). Additionally, recent research on patient-centerd rehabilitation is closely tied with the concept of flourishing. Cogan (2016) notes that in order to promote unique capabilities, goals and potential in those living with ABI, clinicians must provide individualized care in which treatment is collaborative between the provider and the recipient. Such care focused on understanding patient’s lives, their relationships with others and the way in which they want to live. In line with the goals of positive psychology, this research emphasises a shift toward focusing on wellness instead of illness.

Theoretical frameworks of wellbeing and evidence from the field of Positive Psychology and its application through Positive Psychotherapy suggest that people with chronic conditions may benefit from interventions that not only focus on symptom reduction, but also on creating a context for wellbeing. For example, it has been well established that people living with ABI are often socially isolated, with fewer positive relationships, fewer opportunities for meaning and lower community integration (Barber et al., 2018). Thus, increasing opportunities for wellbeing in the ABI population – including enhancing opportunities for positive social connections, developing meaning and purpose in life, and community engagement – has much potential to improve people’s lives.

That said, there is a paucity of research investigating the promotion of wellbeing in the context of ABI. Group and one-to-one positive psychotherapy (PPT) have been reported to increase happiness (Andrewes et al., 2014; Cullen et al., 2018) and reduce symptoms of anxiety (Cullen et al., 2018). These studies reported that the feasibility and effectiveness of PPT in this population is promising. Bertisch et al. (2014) showed a relationship between key constructs in positive psychology (character strengths, resilience, and positive mood states) and factors affecting rehabilitation outcome (i.e., perception of functional abilities post-injury and beliefs about treatment) for people with ABIs. These factors affecting rehabilitation outcome are important in determining treatment success and quality of life outcome in rehabilitation settings and could potentially be impacted on by positive psychotherapy.

Our study builds on these initial promising findings and emerging evidence base by reporting on the findings from detailed qualitative evaluation of the perceptions of people living with ABI following group-based positive psychotherapy. The research question of this study was as follows: Is it possible to build wellbeing in people living with ABI through group-based positive psychotherapy? We review mini-group and interview data against the background of the prevailing approaches that span subjective and psychological wellbeing.

## Materials and Methods

### Ethical Considerations

Service evaluations conducted in the United Kingdom are excluded from ethical review under official policy (GAfREC 2.3.12)^1^. The United Kingdom-based Health Research Authority online decision-making tool confirmed that ethical review was not required^2^. Service evaluations are characterized by minimal risk and therefore fall outside the remit of research ethics committees in the United Kingdom. This exemption was subsequently confirmed by our Research and Development Officer in Swansea Bay University Health Board, provided that data is anonymized or pseudonymized when writing up results for publication.

### Design

The service in which the study took place has historically adopted a participatory approach to service development and evaluation. Service users are involved at a transactional level in the implementation of new initiatives focusing on wellbeing. The present study investigated the experience of service users who completed an 8-week Positive Psychology Intervention (PPI) run in 2017, offered by a community neurorehabilitation service in a major hospital located in South Wales, United Kingdom. A qualitative evaluation (QE) design was employed (Tayabas et al., 2014) to gather in-depth accounts of service user’s experience of the PPI, consistent with United Kingdom National Health Service requirements of evaluating services and patient experience, in keeping with a participatory and context-sensitive approach. QE designs are used to evaluate programmes or services and can employ diverse approaches, including participatory action and interpretative methods, focused on understanding processes in context.

Our study utilized Thematic Analysis (TA) since it allows for large amounts of data collected from multiple participants in naturalistic settings to be analyzed and synthesized into meaningful accounts (Braun and Clarke, 2006). One advantage of thematic analysis is that it is not wedded to any epistemological frameworks and therefore provides flexibility in application. In line with our QE design, a critical realist epistemological perspective was employed in the present study (Bhaskar et al., 1998; Archer et al., 2013). According to this approach, knowledge or “truth” may be shared across individuals, although each individual will have their experience and perspectives, which will be dependent on context.

In line with overarching aims, context and the critical realist approach of the study, we draw on the quality frameworks as suggested by Yardley (2000), which includes four characteristics of good qualitative analysis; sensitivity to context, commitment and rigor, transparency and coherence, and impact and importance.

### Participants

Participants were initially contacted face-to-face by the clinician from a Community Neurorehabilitation Service (CNS) based in South Wales, after which letters were sent including details relating to time, date and location of the PPI. A total of 19 participants were purposively invited to attend the course and 13 accepted the invitation. Reasons for not accepting the invitation included other commitments, employment, travel difficulty, and surgery. Once the course had begun, there were no dropouts. Four out of the 13 participants had attended a course previously, and were subsequently invited to become mentors. These individuals were trained by a member of the clinical team to deliver some of the practical aspects of the intervention and asked to share their own lived experiences of using some of the PP techniques that had learnt. One participant was lost to follow up due to being discharged from the CNS. Details of the twelve participants who participated in this study are included in Table 1. Participants are referred to as pseudonyms in the manuscript, categorized according to mentor or participant status, followed by an identification number (e.g., “M1,” “P1”).

**TABLE 1:** Participant characteristics.

	**Sex**	**Age**	**Injury**	**Comments**
M1	Female	53	Inflammatory idiopathic left limbic encephalitis	M1 was interviewed with 3 other mentors in a mentor-only mini group interview
M2	Female	62	Moderately severe traumatic brain injury	M2 was interviewed with 3 other mentors in a mentor-only mini group interview
M3	Female	60	Suspected encephalitis	M3 was interviewed with 3 other mentors in a mentor-only mini group interview
M4	Female	50	Mild Traumatic Brain Injury	M4 was interviewed with 3 other mentors in a mentor-only mini group interview
P1	Male	68	Severe traumatic brain injury	P1 was interviewed in mini group for participants along with P2, P3, P4 and P5.
P2	Female	53	Moderate traumatic brain injury	P2 was interviewed in mini group for participants along with P1, P3, P4 and P5.
P3	Male	58	Severe traumatic brain injury	P3 was interviewed in mini group for participants along with P1, P2, P4 and P5.
P4	Male	29	Moderate to severe traumatic brain injury	P4 was interviewed in mini group for participants along with P1, P2, P3 and P5.
P5	Male	55	Severe Traumatic brain injury	P5 was interviewed in mini group for participants along with P1, P2, P3 and P4.
P6	Female	73	Moderate traumatic brain injury	P6 was interviewed individually using a face-to-face interview with an assistant psychologist who had been present for some of the group sessions.
P7	Female	34	Suspected hypoxic brain injury	P7 was interviewed individually using a face-to-face interview with an assistant psychologist who had been present for some of the group sessions.
P8	Female	37	Cerebellar infarct following a traumatic artery dissection.	P8 was interviewed individually using a face-to-face interview with an assistant psychologist who had been present for some of the group sessions.

All participants had a confirmed diagnosis of ABI, had experienced psychological difficulties and/or reduced social participation and had received rehabilitative treatment before participating in the study. The recruitment process was pragmatic in nature and involved the clinician selecting individuals whom she had worked with from within the CNS. The clinician exercised judgment relating to participants’ capacity to benefit from the course, as well as the likelihood of disrupting the learning opportunity for others. The decision process was centerd around the following inclusion and exclusion criteria.

Inclusion criteria included age greater than 18; confirmed diagnosis of ABI; ability to actively engage in the intervention as determined by their clinician; living in the community; evidence of psychological difficulties during the initial clinical interview when patients access the brain injury service; living within the catchment area of the participating health boards; at least 3-month post-injury at recruitment, allowing time for spontaneous recovery and for the person to become aware of their difficulties and the implication of these on their lives. Exclusion criteria including receptive or expressive language difficulties, or extremely low memory function that may preclude people from engaging meaningfully; medical or psycho-social reasons, based on risk assessments by the referring clinician; potentially disruptive to other group members as determined by their clinician; not able to provide informed consent. Mentors were subject to the same inclusion criteria as participants with the exception of showing “evidence of psychological distress.” Mentors were also subject to the following additional inclusion criteria: demonstrated ability to be responsive and sensitive to the needs of others; known to and recommended by their referring clinical team; good interpersonal skills; willing and able to commit to training as well as attending all eight PPT sessions.

### Intervention

The positive psychology course was developed in partnership between clinical and academic psychologists. The course was delivered in a semi-structured format and was designed to incorporate aspects of subjective wellbeing (Diener, 1984; Fredrickson, 2001), psychological wellbeing (Ryff, 1989) and PERMA theory (Seligman, 2011; Table 2). Participants were given a resource booklet which outlined the structure of the course as well as pages for note-taking and completion of written activities. The course was structured to include psychoeducation and practical elements, allowing participants to engage in a range of activities designed to improve wellbeing after being provided with relevant background and context. While the clinician ensured that all course content was delivered, open discussion was encouraged. The semi-structured nature of the course promoted a transactional approach between the clinician delivering the course and participants. Discussions enabled participants to share their own stories relating to their experience of ABI, facilitating group bonding and positive social ties. Sessions were typically 2 h long with a break in the middle. Additional breaks were given if required.

**TABLE 2:** Structure of positive psychotherapy intervention.

**Week**	**Focus**	**Comment**
1	Introduction to positive psychology	This session focuses on introducing participants to the course. They are given an informational booklet outlining the course structure which also has worksheets and note-taking spaces, for each session. Models of wellbeing are taught including; hedonic, eudaimonic and PERMA. Participants are asked to complete a “wheel of wellbeing” which outlines the 5 pillars of the PERMA model to encourage participants to think about how these relate to their own lives.
2	Relaxation	This session focuses on learning about the role and function of stress and anxiety and learning how to activate the relaxation response using exercises of relaxation techniques, such as diaphragmatic breathing and guided imagery. Participants are given CD’s with guided relaxations including a loving kindness exercise which encourages participants to think about and feel love for important people in their lives. Participants complete a guided imagery exercise of a safe place.
3	Character Strengths	Participants complete the VIA character strengths and explore their top strengths (Peterson and Seligman, 2004). They write examples of when they have used them in their lives prior to the course and think of new ways to mobilize them in future.
4	Positive emotions	Participants are to learn the role and function of emotions and explore negativity bias’ and the benefits of increasing frequency of positive emotional experiences. Participants complete a gratitude exercise; participants note down three positive things they are grateful in their day-to-day lives. Participants are encouraged to complete this exercise daily over the course of a week and take note of this in their information booklet. Participants are encouraged to complete one random act of kindness a day and record this exploring Barbara Fredrickson’s Theory of love in terms of shared experiences to facilitate positive emotions, bio-behavioral synchrony and mutual care and concern. Participants then experience a loving-kindness meditation.
5	Optimism	Participants learn about optimism, learned helplessness and learned optimism. Participants learn how to alter thinking styles using CBT to adopt a more optimistic outlook. Participants complete activities relating to the ABCDE model of optimism.
6	Mindfulness	Participants learn about the four key elements of mindfulness according to Hölzel et al. (2011) and how to apply these to everyday life. Participants complete two exercises at home as instructed in their workbook; to use mindfulness when eating and mindful meditation. Mindful meditation. Participants then complete a grounded exercise which evokes the five senses when meditating.
7	Connection between body and mind	Educational information on sympathetic and parasympathetic nervous system and responses to antecedents are delivered to participants as well as discussions on the effects of chronic stress. Participants are taught about the vagus nerve, heart rate variability and their connections to health outcomes. Positive health behaviors are explored. Participants then complete exercises to increase HRV e.g., exercise followed by deep breathing with prolongation of the outbreath.
8	Making positive changes	Participants learn about habits and how to move away from bad habits, to good habits. Willpower and the path of least resistance is explored and reducing activation energy for desired behavior. Participants are invited to discuss how they can apply these principles to their own lives.

### Data Collection

Semi structured interviews were carried out across two separate mini-groups, one for the mentors (*n* = 4) and the other for participants (*n* = 5). Three additional individual (*n* = 3) interviews were separately conducted to accommodate those individuals who requested that they be interviewed separately. All interviews were conducted in a hospital, within a Community Neurorehabilitation Service where the intervention had taken place, following the positive psychotherapy intervention. Participants were initially invited to attend the interviews face-to-face at the end of the course, subsequently letters were sent to participants detailing the time, location and date. All interviews were conducted by a single Assistant Psychologist (AP) (female) with postgraduate training in psychology. The AP had established relationships with the participants over the 8-week period by attending groups and greeting participants when they visited the clinic. During interviews, only the AP and the participants were present; clinical staff (ZF, HB) were outside of the room, but nearby for governance reasons. Field notes were made during interviews by the AP to include overall impressions of perceived improvements to wellbeing according to individual representation and possible improvements to future services. These notes were then passed to the leading clinician to guide future interventions.

Before the interviews began, participants were informed of the purpose of the interviews and that anonymized data may be used for evaluative purposes. Verbal consent was then obtained. Each interview followed a similar pattern, beginning by exploring the initial hopes of the group versus experiential gains, inviting individuals to give feedback regarding the group and its delivery, commenting on salient aspects and whether the course had impacted upon future recovery. Questions for participants were open-ended and were developed iteratively over the course of the discussions, consistent with the need to be flexible and responsive to material in qualitative research. The interviewer sought to promote group discussion by asking others if they agreed with the points that were raised and by asking participants to engage in discussion.

All interviews were recorded using a “Voice Memos” application. Verbal consent was gathered from all participants for the recordings to be transcribed and analyzed. Audio data of interviews totaled 3 h and 1 min. Interviews were 36 min in duration on average and ranged from 20 min to 1 h and 3 min. The interview audio files were then transcribed orthographically so that the true essence of the data would be captured by incorporating utterances such as “ah, um etc.,” as well as utilizing grammatical correctness to indicate pauses, end of statements and exclamations. Interviews were transcribed verbatim except for the names of participants, staff names and locations, which were omitted to ensure anonymity.

### Data Analysis

The software ATLAS. TI was used to manage the data at the coding stage, after which established codes were organized into categories and then managed using Microsoft Excel. All categories were then referenced against original codes in ATLAS to check for consistency and appropriateness of categorization. The analysis of the data followed a guide introduced by Braun and Clarke (2006), which outlines a 6-step procedure to good Thematic Analysis (see Table 3).

**TABLE 3:** Braun and Clarke (2006) 6-step Guide to Good Thematic Analysis.

**Phase**	**Examples of Procedure for Each Step**
1. Familiarization	Transcribing data: reading and re-reading; noting down initial codes
2. Generating Initial Codes	Coding interesting features in the data in a systematic fashion across the data set, collating data relevant to each code
3. Searching for Themes	Collating codes into potential themes, gathering all data relevant to each theme
4. Involved Reviewing Themes	Checking if the themes work in relation to the coded extracts and the entire data-set; generate a thematic map
5. Defining and Naming Themes	Ongoing analysis to refine the specifics for each theme; generation of clear names for each theme
6. Producing the Report	Final Opportunity for Analysis selecting appropriate extracts; discussion of analysis; relate back to the research question or literature; produce report

Braun and Clarke (2006) note that qualitative research will inevitably involve a mixture of both inductive and deductive approaches. In line with the quality criteria set out by Yardley (2000), coding applied “bracketing” to maintain critical awareness of the basis for sense making, and allowed the researcher to choose to focus on use of positive psychology theory in the data, or to bracket or suspend this in order to maintain sensitivity to the meanings and experiences of participants (Tufford and Newman, 2012). Data was not coded by a second coder because – in line with, Braun and Clarke (2006) – measures of inter-rater reliability between multiple coders simply reflect the degree to which coders follow the same procedure, rather than accuracy of the coding process, *per se*.

## Results

Recurrence of themes was noted during analysis, and participants’ experiences could be coded within a common or shared set of themes and sub themes (Tables 4, 5).

**TABLE 4:** Themes and Subthemes for Thematic Analysis for non-mentor participants.

**Theme**	**Subthemes**
Empowerment	Achievement
	Confidence
	Sense of transformation
Social Opportunity	Relatedness with others (ABI and non-ABI)
	Understanding the self through others
**Coping**	
**Cultivating Positive Emotion**	
Barriers	Stage in recovery
	Location/Environment of intervention

**TABLE 5:** Themes and Subthemes for Thematic Analysis for Mentors.

**Theme**	**Subthemes**
Empowerment	Meaning through providing support Achievement
	Feeling Valued
	Confidence
Social Opportunity	Relatedness with others (ABI and non-ABI)
	Helping others
Coping	Reframing
	Mobilization of character strengths
**Consolidation of skills**	
**Cultivating Positive Emotion**	
Barriers	Stage in recovery
	Location/Environment of intervention

For non-mentor participants, Thematic Analysis (TA) identified the following themes: (1) Empowerment; (2) Social Opportunity; (3) Coping; (4) Cultivating Positive Emotion; and (5) Barriers (see Table 4).

For mentors, TA identified the following themes: (1) Empowerment; (2) Social Opportunity; (3) Coping; (4) Consolidation of skills; (5) Cultivating Positive Emotion; and (6) Barriers (see Table 5).

**FIGURE 2:**
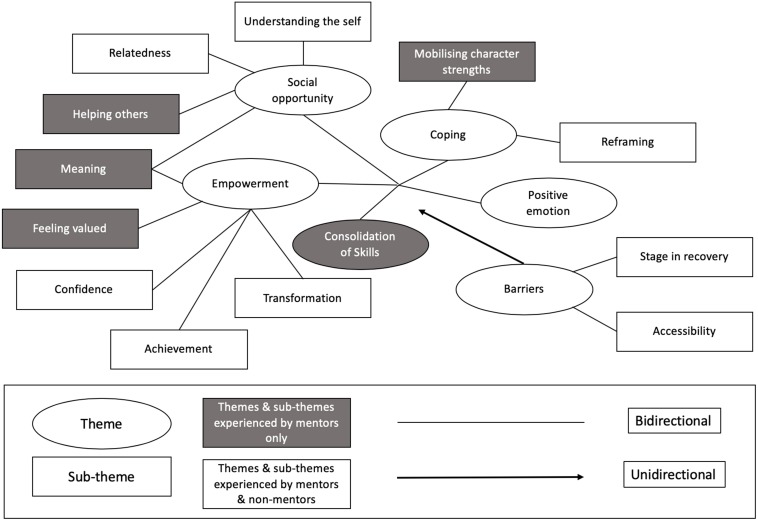
Visualization of themes and sub-themes identified in thematic analysis of transcripts collected during focus groups and interviews on people living with ABI following positive psychotherapy.

Themes for mentors and participants were then converged onto a single thematic map (see Figure 2). This convergence process was achieved by collapsing identical themes and then converging respective sub-themes. Links between themes were then included to the relationships between themes and sub-themes.

A full description of themes and sub-themes is provided in Supplementary Materials, while the reported results below have been shortened, describing only the main themes and observations, for brevity.

### Empowerment Theme

This theme captured an overall sense of empowerment experienced by both mentors and participants. A combination of enhanced confidence; sense of achievement; feeling valued by others, gaining meaning by helping others and a sense of transformation all contributed to an overall sense of empowerment. Achievements such as gaining new skills and overcoming challenges, facilitated a sense of independence and control over personal circumstances.

“*I’d lost my husband in the supermarket and I didn’t have a panic attack. I just didn’t move from where I was, I just waited for him to come back to where he last saw me. And then I said to him*…*I said no I’m going to go shopping on my own and it was a huge, huge thing*… *I came out and I must have felt about ten foot tall.*” (P6).

Individuals became more resourceful and welcomed situations in which they would be able to further develop confidence, such as speaking to members of the public.

“*its given me more confidence to try things that I wouldn’t have had the confidence to do before. I would have automatically gone into something thinking I can’t do that and now I’m sort of open to try things. And I’ve tried a lot of things. Most of them haven’t worked, but you know, I’ve tried them*” (M3).

People living with ABI were eager to give back to their community by helping others, an experience that was facilitated by participating in the positive psychotherapy group as mentors. This opportunity to help others by drawing on their experience of living with an ABI gave them meaning and purpose by feeling valued and by adopting the view that their purpose within the intervention was to provide support and hope for positive change.

*“when I am mentoring, I do feel valued. I feel somebody, you know, I’ve got a bit of self-worth again, which you tend to lose”* (*M1*).

Mentors remarked that their purpose is to make others feel safe and valued;

*“It’s about making them feel safe and valued”* (*M1*).

Mentors had experienced positive change themselves through completing the course. This positive change was reflected with a sense of transformation described by both mentors and participants, often explaining that their life had changed for the better and that personal development was evident through gains in ability, implementation of coping strategies, increased self-efficacy, confidence and acceptance of injury.

*“I went in thinking that can you really learn happiness, and I learnt about positivity*… *and to walk away as a happier person, I mean you can’t give a medicine for that”* (*P8*).

### Social Opportunity Theme

This theme captured participants sense of better understanding of the self, relatedness with others, and for mentors, the sense that they were helping others. Participants explained that having a shared understanding of ABI facilitates a comfortable, judgment-free and acceptance-based atmosphere, which in turn promotes of sense of unity based on equality;

*“I think if you want to express something you can talk to somebody here and they can understand it. And they don’t judge you*…*”* (*P1*)*; “Everyone comes in at the same level”* (*P3*).

Mentors were viewed as inspirational, as participants felt that they had made significant progress in their recovery;

*“Yeah it’s exactly the same with the mentors as well. You can see them and say ‘right okay the level of improvement they have made and [see what] we can get to”* (*P4*).

Participants described that group membership allowed them to explore their individual symptomology. Participants were able to explore confusing and difficult behavior in a comfortable social arena;

“*I couldn’t explain myself before, whereas now I have outbursts of where I have got no breaks of*… *before I would say things and I would get so upset that I wouldn’t get over it, why have I done that? I didn’t understand why I was doing it. And I sort of isolated myself then. Sort of punishing myself. Whereas now I think it’s okay to have that blip*.” (*P2*).

### Coping Theme

This theme captures the sub-themes of positive reframing and mobilization of character strengths. Coping strategies learned during the intervention included grounding techniques, gratitude techniques, and humor;

“*I am a lot happier, and because I have gone back to basics and hearing other people’s stories at how they appreciate just the simple things you know*… *I’m putting a positive slant on things rather than a negative slant and I’ve been trying to do that a lot more.*” (P8).

The ability to reframe thoughts led to individuals viewing anxious situations as a marker for challenge;

*“everybody has to fight their own battle, but you can get there and I think you can be happy no matter what.”* (*P8*)*;*

Coping mechanisms such as reframing were underpinned by mobilization of character strengths (for mentors). The shift in attention from negative to positive emotion was reinforced by recognizing strength, instead of weakness;

“*think when you’ve had an illness, and like us with the brain damage, afterward everything is negative. Because you’re focusing on what you used to do and you can’t do it anymore. But then you read your strengths and you think ‘oh hang on a minute, you know*” (M2).

### Cultivation of Positive Emotion Theme

Positive emotions were cultivated when experiencing empowerment, social opportunity, coping and consolidation of skills. For example, achieving the ability to execute tasks was associated with happiness and joy;

*“I sat in the car and had this huge great big grin on my face*…*I didn’t want to go I just felt so so amazing, so happy, I literally I felt so happy because to me I had achieved something”* (*P2*).

The creation of a new social network and social participation also lead to an increased sense of happiness;

“*the friendships you make, and to walk away as a happier person*” (*P8*).

Helping others was associated with positive feelings;

“*it’s helped me put it into perspective and it sounds awful but that made me feel good*” (*M3*).

Positive emotions were also used as a coping mechanism when dealing with difficult situations;

“*sometimes I may laugh at something I done whereas before I would cry*.” (*P2*).

### Consolidation of Skills Theme

Mentoring allowed individuals to reinforce knowledge, serving as a reminder to maintain skills;

“*I’d started to only see the negatives again. Whereas this has helped me to see the positives as well as the negatives and then deal with the negatives then, isn’t it*…*It’s like layers, like I’m building upon layers and then I think oh yeah, I remember now. I almost needed it [positive psychotherapy] to consolidate the information myself.*” (M2).

Revisiting the course several times over through mentoring also allowed mentors to absorb new information that they believed they had missed during prior courses;

“*You just get little nuggets of information. Like somebody will say, just as a throwaway line, and you’ll think- what? And you say, sorry can you repeat that? And you think- Ah! I forgot all about that! Why aren’t I doing that? Um so I just get as much from it, well, probably more.*” (M4).

### Barriers to Efficacy Theme

These theme captures two sub-themes including ‘stage in recovery’ and accessibility issues’. One participant (P7) noted that her stage in recovery impacted on how beneficial she found the group. She commented;

“*I think if I’d have had this 5 years ago, 4 years ago, even 2 years ago when I was at rock bottom. I think it would have been such a great help*…” (P7).

However, P7 explained that although she felt further along in recovery and part of the course was less relevant to her, she still found it helpful to compare her own progress with others. In that sense, she felt grateful for her own progress;

“I feel further along with it. And I felt that even though it was really good for me to see that actually I’m not as bad as I think, other people.”

Many participants noted that the location of the intervention was difficult to access. The course was not located in proximal locations for some, which meant traveling long distances to participate in the course. Participants further noted that parking facilities at the location were extremely limited. Even with disabled badges, parking was still considered to be an issue;

*“she had a disability badge and she still wouldn’t be able to park so well*… *parking was a major thing there.”* (*P7*).

## Discussion

This study explored whether positive psychotherapy might promote wellbeing in people living with ABI, and examined participants’ experience of the intervention against the background of prevailing theoretical models. Thus, during analysis, concepts relating to these wellbeing models were identified and subsequently organized into themes within the context of ABI. The six themes identified in this paper included; empowerment, social opportunity, coping, consolidation of skills, cultivation of positive emotion and barriers to the efficacy of positive psychotherapy.

In line with Subjective Wellbeing (SWB; Diener, 1984; Fredrickson, 2001), Psychological Wellbeing (PWB; Ryff, 1989) and PERMA theory (Seligman, 2011), results indicate that positive psychotherapy promoted wellbeing in people living with ABI. Elements of both eudaimonic and hedonic aspects of wellbeing were identified in the analyzed transcripts. Consistent with eudaimonic theories of wellbeing, participants derived a sense of purpose and meaning through course participation. Mentors characterized their purpose as helping others by providing hope for future recovery as well as providing a unique perspective of ABI that only comes with lived experience. Participants derived purpose and meaning by developing skills and abilities, with an eye toward personal recovery so that they could eventually help others in similar situations.

Consistent with hedonic theories of wellbeing, the cultivation of positive emotion was associated with experiencing positive emotions due to learning and applying new skills, overcoming challenges, building and strengthening social ties and recognizing progress in one’s recovery. Moreover, participants used coping skills to manage negative emotions by accepting them and refocusing attention on positive elements, suggesting that they had achieved greater emotional control. Central to SWB theory, emotional control led to the experience of low negative affective and high positive affect, promoting a sense of wellbeing. Consistent with Fredrickson’s (2001) broaden and build theory, and Seligman’s PERMA model (2011), a sense of achievement was usually accompanied with positive emotion, which spurred individuals to seek more situations in which they could experience the positive emotions associated with achievement, thus broadening their thought-action repertoire.

Factors associated with Ryff’s Psychological Wellbeing theory (Ryff, 1989) such as self-acceptance, personal growth, purpose, environmental mastery and positive relations; and Seligman’s PERMA model (2011) were discussed by participants. For example, participants experienced improvement in abilities, such as communication skills and the ability to overcome challenges, deriving a sense of achievement when doing so. A sense of achievement was associated with increased independence and autonomy for some. Gains in ability to execute a task resulted in reduced need for support. Participants described positive life changes due to personal development including gain in abilities, the development and employment of coping mechanisms, recognition of improvement and behavior over time and alterations in beliefs and attitudes toward challenges. The theme of “Social Opportunity” captured the positive relationships that participants experienced where they described feeling reciprocal support and empathy from others.

Thus, in terms of the aforementioned wellbeing theories, positive psychotherapy enhanced wellbeing for people living with Acquired Brain Injuries (ABI). In addition, this study also outlined context-specific factors that contributed to enhancing wellbeing. For example, within the theme “Social Opportunities” positive relationships were fostered leading to a sense of togetherness and a shared experience of living with ABI. Sharing of information related to symptoms, recovery and support, facilitated group bonding and strengthened relationships between participants. These findings are in line with other research on relatedness and ABI which suggests that a sense of belongingness is associated with psychosocial wellbeing (Bay et al., 2002, 2012). Individuals noted that the sense of relatedness was a core feature of the intervention as they described a sense of misunderstanding and stigmatization from individuals living without brain injury. Research has previously demonstrated that stigmatization of brain injury is reflected in negative attitudes toward survivors (McLellan et al., 2010). In addition, research has shown that the adverse effects of brain injury, such as anxiety and speech difficulties, are compounded by people’s misunderstandings of brain injury as individuals attempt to minimize perceived mistakes leading overcompensation or societal withdrawal (McClure et al., 2006). Participants felt that their actions would not be wrongly judged by others in the group, providing them with a safe and comfortable space in which to explore their authentic self. In turn, participants described “feeling okay” to act outside of their comfort zone, promoting further opportunity for growth.

In addition, individuals in this study described learning and employing various coping skills. Participants found coping strategies to be beneficial when experiencing fatigue, a common symptom of ABI (Belmont et al., 2006). Research has demonstrated that the development of coping skills can help people with ABI adopt a more active lifestyle, as they learn strategies to manage the demands of the environment (Tomberg et al., 2005). The coping skills developed over the course of positive psychotherapy helped participants to reconcile loss relating to ability, social network, social participation and identity. Participants describe employing acceptance-based strategies, such as gratitude techniques and mindfulness, when becoming frustrated or distressed with loss. Through reframing, participants were able to focus on the positive aspects of one’s life, giving rise to a new sense of appreciation for one’s residual abilities. This process of reframing and accepting loss is consistent with Gracey et al. (2009) “Y-Shaped” model. According to this model, the process of adaptation and reintegration into society following brain injury involves identifying, understanding and resolving social and psychological discrepancies. These discrepancies can include a lack of understanding from others or stigmatization of ABI, withdrawal from social participation, and identified differences between pre-injury and current sense of self. As an individual works to resolve these discrepancies, aspects of continuity of self are discovered and developed leading to new, adaptive meanings associated with the self.

Consistent with the “Y-Shaped” model, the present study found that mentors were able to mobilize character strengths to underpin coping efforts. The sub-theme “Mobilization of Character Strengths” denotes how mentors first identified their character strengths, then understood how to employ them after which strength mobilization helped to create a more meaningful sense of identity. For example, mentors reported knowing themselves better and feeling more adaptive to situations. Mentors were then afforded the opportunity to consolidate their skills revisiting the course and absorbing new information. In addition, mentors consolidated their sense of meaning, to help others and provide hope for the future, with each course.

The theme relating to barriers outlined some of the issues raised by participants that may impact on the efficacy of the intervention. Relating to the sub-theme “stage in recovery” one participant in particular, noted that she felt further along in her recovery than her peers, resulting in frustration with elements of the intervention. She believed that the discussion of negative emotion, such as depression and anxiety in a group setting, did not facilitate positive change, instead viewing this as an example of “emotional dwelling.” The frustration experienced early in the intervention resulted in reduced participation with other aspects of the course, such as the session on mindfulness. She explained that her reduced participation was a result of not wanting to dwell on negativity. Sessions were semi-structured, allowing for user-led discussions, so that participants could explore ideas salient to them. Holding the view that she was further in recovery, she noted that she did not relate to these ideas at her stage of recovery. However, this participant also described several positive experiences including comparing her progress to others who were less advanced in their recovery. According to social comparison theory (Festinger, 1954) downward social comparison is used as a means of self-evaluation, to compare the self with others that are considered to be worse-off to feel better about the self or personal circumstances (Tesser et al., 1988). This individual also noted that she had made a close friend in the group who was regarded as being at her level of recovery and described feeling a sense of relatedness and self-esteem as the only outcomes of the intervention for her. This suggests that group facilitators need to consider perceived stages in recovery in future psychotherapy and to group individuals according to their preferred stage when possible.

Another barrier was captured by the subtheme of “accessibility and location.” Several participants noted that it was difficult for them to access the course, often traveling several hours each way in attendance. In addition, several participants described a lack of service provision in their local area, noting that accessing any type of community care-based therapy is difficult. Whilst, this issue did not translate to drop-outs in the present study, the mention of accessibility was recurrent, suggesting it was a salient concern for participants. The lack of service provision and long travel distances reflects the so-called treatment gap and lag (Wang et al., 2004; Patel et al., 2010), highlighting a need for major reform of current mental health treatment and its availability, especially when considering ongoing challenges associated with the increasing burden of chronic disease (GBD 2013 Mortality and Causes of Death Collaborators, 2014; Vos et al., 2015). Treatment gap refers to the numbers of people needing treatment and not receiving it, while the treatment lag refers to the amount of time taken to receive care when it does exist. Studies suggest that the mental health treatment gap exceeds 50% in all countries, and 90% in those countries with less resources (Patel et al., 2010), while the treatment lag can be as long as 10 years (e.g., Wang et al., 2004).

Recent criticism of wellbeing models has emphasized their individualistic focus and a lack of consideration of community, the environment within which individuals live, wider societal influences and socio-structural factors such as community resources and inequality (e.g., Carlisle et al., 2009; Ehrenreich, 2010; Davies, 2015; Frawley, 2015). Unfortunately, this has led to a tired debate between proponents of individualist versus structural approaches to health promotion, ignoring a need for their combination. In response, we have proposed a model of health and wellbeing, the GENIAL model, that spans both approaches (Kemp et al., 2017; Mead et al., 2019), emphasizing a role for the individual, community and the wider environment. Our model and its recent iteration (Kemp et al., 2017; Mead et al., 2019) characterizes socio-structural influences over individual health-related behaviors and subsequent wellbeing, emphasizing an important role for community cohesion and collective efficacy to support individual health-related goals. In doing so, our model emphasizes important roles for novel vehicles for change such as task shifting and partnership working to improve individual wellbeing and longevity. Further research is also needed on the role community organizations can play in the health and wellbeing of people living with ABI to support interventions such as positive psychotherapy.

With respects to limitations, the study was based on qualitative analysis of a service-user evaluation, and conclusions are therefore restricted to the service from which data was collected. However, results are interpreted in the context of available theory and therefore lay useful foundations for healthcare service improvement. Data collection was also restricted to a single timepoint as it was not pragmatic to carry out repeat interviews. One advantage of repeat interviews is the ability to explore processes over time. Whilst this will be important for future work, our goal was to identify the factors that contribute to wellbeing in participants and mentors living with ABI. Future studies should consider exploring how these processes change over time. Similarly, when conducting interviews, it was not feasible or pragmatic to return transcripts to participants for comment. However, our service is underpinned by a participatory and collaborative approach to service development and improvement. We note further that findings have been shared with and positively received by the brain injury community, demonstrating enthusiasm for improving the healthcare of people living with ABI by focusing on wellbeing rather than a restricted focus on impairment. Another limitation is relatively small sample size, which comprised those who access the service, as well as subsequent inclusion and exclusion criteria. However, recurrence of themes was noted during analysis, and participants’ experiences could be coded within a common or shared set of themes and sub themes. We further note that there has been debate over application of the principle of saturation by qualitative researchers who have recognized that complete saturation is rarely possible (Charmaz, 2006) and may not always be appropriate (O’Reilly and Parker, 2012; Malterud et al., 2016). Finally, it is recognized that the use of mini-groups and individual interviews are distinct tools, however we adopted a mixed approach to ensure that the needs and preferences of all service users were met. Carlsson et al. (2007) note that those with ABI are often excluded from qualitative enquiry due to the methodological challenges incurred from communication issues or fatigue. As such, it is recommended that individuals with ABI are supported throughout the interview process. In this case, we sought to provide individuals with a choice over interview type in order to provide more individualized communication support. In this way, we have followed the guidance of Carlsson et al., and employed adjustments to our methodological approach in order to capture the voices of individuals who are otherwise marginalized (Paterson et al., 2001).

In summary, this study provides new qualitative data to support the use of positive psychotherapy for enhancing wellbeing in individuals living with ABI. Importantly, and for the first time, certain themes (i.e., consolidation of skills) and sub-themes (helping others, meaning, feeling valued and mobilization of character strengths) were identified, emphasizing the benefits associated with acting as mentors for those experiencing positive psychotherapy. These findings suggest that mentors may provide the healthcare sector with an underutilized community resource for ensuring that mental health services for people living with ABI are more sustainable and may help to bridge the treatment gap and better support service users including mentors and participants at the same time. Further research exploring these novel insights is warranted. Critically, our findings indicate that it is possible to improve the wellbeing of people with ABI, despite the impairments caused by their condition. In terms of healthcare, this suggests a need for more effective models of care for people with chronic conditions (including ABI) which not only focus on reducing impairment, but also on improving wellbeing. Given, emerging evidence that health and wellbeing is contingent on individual, community and environmental factors (see Mead et al., 2019 for review), “health and wellbeing” should no longer be thought of as “the remit” of the health service. There is a need for greater partnership working between health services, universities and community organizations to create evidenced-based environments designed to improve health and wellbeing.

## Data Availability Statement

The datasets generated for this study are available on request to the corresponding author.

## Ethics Statement

Ethical review and approval was not required for the study on human participants in accordance with the local legislation and institutional requirements.

## Author Contributions

ZF and AK conceptualized, planned and supervised the project supported by JP, FG, and JT. ZF was responsible for delivery of the intervention, supported by AK, HB, and LW. CT completed this study in partial fulfilment of her MSc degree. All authors edited the manuscript for intellectual content and approved the final version prior to submission.

## Conflict of Interest

The authors declare that the research was conducted in the absence of any commercial or financial relationships that could be construed as a potential conflict of interest.

## Footnotes

1 https://tinyurl.com/rcp2cqe

2 https://tinyurl.com/wdrydgg
