# Supplementary material for: Building Wellbeing in People With Chronic Conditions: A Qualitative Evaluation of an 8-Week Positive Psychotherapy Intervention for People Living With an Acquired Brain Injury
Source: Front Psychol. 2020 Jan 31;11:66. doi: 10.3389/fpsyg.2020.00066 (PMC7006056; doi:10.3389/fpsyg.2020.00066)
Supplement: Supplementary file 1 [file Table_1.DOCX]

**Empowerment Theme**

This theme captured an overall sense of empowerment experienced by both mentors and participants. A combination of enhanced confidence; sense of achievement; feeling valued by others, gaining meaning by helping others and a sense of transformation all contributed to an overall sense of empowerment. Achievements such as gaining new skills and overcoming challenges, facilitated a sense of independence and control over personal circumstances. Individuals became more resourceful and welcomed situations in which they would be able to further develop confidence, such as speaking to members of the public. People living with ABI were eager to give back to their community by helping others, facilitated by participating in the positive psychotherapy as mentors. This opportunity to help others by drawing on their experience of living with an ABI gave them meaning and purpose by feeling valued and by adopting the view that their purpose within the intervention was to provide support and hope for positive change. Mentors had experienced positive change themselves through completing the course. This positive change was reflected with a sense of transformation described by both mentors and participants, often explaining that their life had changed for the better and that personal development was evident through gains in ability, implementation of coping strategies, increased self-efficacy, confidence and acceptance of injury.

*Achievement sub-theme*

Group members described feeling a sense of achievement in relation to gaining the ability to execute a task successfully and independently. P6 explained that she was unable to independently shop and relied on her husband to both complete the task and offset panic attacks. After participating in the group for 4-5 weeks, this participant was able to independently shop without the assistance of her husband;

“*I’d lost my husband in the supermarket and I didn’t have a panic attack. I just didn’t move from where I was, I just waited for him to come back to where he last saw me. And then I said to him…I said no I’m going to go shopping on my own and it was a huge, huge thing… I came out and I must have felt about ten foot tall.*” (P6)

P6 goes on to say how this gain in ability to successfully and independently execute a task, one that was previously not possible to fulfil, gave her a sense of achievement;

“*I didn’t want to go I just felt so, so amazing, so happy, I literally felt so happy because to me I had achieved something that was a giant milestone in my recovery, it really was, I mean now I can pop into a shop without having to think about it too much.*”

Similarly, mentors experienced a sense of achievement after helping to deliver the intervention;

“*I genuinely went in thinking, oh, I can’t do this. I really thought that I couldn’t do it ‘cause I hadn’t seen anyone else do it and I thought it would be too much. So then, I did it, which was a sense of achievement*” (M3)

Individuals felt that the intervention enabled them to successfully execute tasks that were previously viewed as unachievable. In doing so, a sense of achievement was developed;

*“it gave me a confidence boost because I had achieved something after so long of not achieving. I had achieved something. So it helped me, even though I was helping other people, it helped me as well.”* (M3)

*Confidence sub-theme*

Group members describe situations in which their ability to perform tasks had dramatically improved, particularly the ability to successfully converse with others. P2 declared:

*“I mean the first time I went… I mean most of you guys were there, I couldn’t even introduce myself.”*

P2 explained that she had made several friends from the group and had contacted them outside of group, suggesting that her ability to converse successfully had improved;

*“I met a lot of friends. And some that I contacted outside of the group because they understood the injury and helped me through a difficult time.”*

Participants explained that group membership afforded them the ability to practice and strengthen communication skills;

*“So yes, it was a good opportunity to speak… well you can hear my speech now it’s very stuttered. But at least I get the opportunity to use my speech. And hopefully improve it.” (P4).*

P3 explained that he had previously had difficulty communicating due to a speech impediment;

*“At the beginning of the group I used to stutter.” P3 continued; “only now I’ve noticed I’ve stopped stuttering altogether.”*

This improvement in communication skills was facilitated by the shared experience of living with an ABI, individuals felt comfortable with one another to explore and share their story.

“*the group taught me that it’s not the end of the world that there is life after having a brain injury, that you can get your confidence back, that you can actually speak up and you can say how you are feeling*” (P6)

A sense of increased confidence enabled participants to feel comfortable to explore communication with others outside of the group. Participants embraced feeling beyond their comfort zone when interacting with new people and attributed such to confidence development within the group.

“*And the taking me out of my comfort zone that I had built up around me has also been really positive because it meant that the next time I went out, … in public as it were… umm I felt more confident because I had been to the group so it made just going out and meeting people just that much easier*.” (P6)

Both mentors and participants were spurred on by improvements and confidence in their ability. Consequently, they sought more situations in which they could further their development. Some participants engaged in new and unfamiliar activities, which they would not have attempted if it weren’t for their newfound confidence born out of group attendance.

“*its given me more confidence to try things that I wouldn’t have had the confidence to do before. I would have automatically gone into something thinking I can’t do that and now I’m sort of open to try things. And I’ve tried a lot of things. Most of them haven’t worked, but you know, I’ve tried them*” (M3).

*Feeling valued sub-theme*

Mentors noted that their sense of self-worth had improved over the course of the intervention;

“*I felt as If I was worth something*” (M3).

Mentors refer to a sense of loss when talking about a revived sense of value. They explain that feelings of redundancy that comes with vocational loss, or loss in societal participation and how that contributes to poor self-worth and a lack of value;

“*it gives you sort of, self-worth as well. You feel valued, cause we’ve all had good jobs and we’ve all had responsible jobs and when that’s taken away, you just feel a bit useless. And you know… this has been marvellous for me*” (M3).

Mentors explained that mentorship creates an opportunity to rejuvenate a sense of self-worth and value;

*“when I am mentoring, I do feel valued. I feel somebody, you know, I’ve got a bit of self-worth again, which you tend to lose.” (M1)*

Mentors believed that they had a unique perspective to offer, that is beyond the scope of clinicians without first-hand experience of ABI. They described a sense of understanding that could be expressed in terms of empathy and acceptance;

“*we come into it on that side of it because we sort of get across, you know, it’s okay if you’re tired. It’s okay if you don’t understand, it’s okay if you don’t want to do all of these things*.” (M3)

Mentors believe that their unique perspective allows them to notice subtleties in the experience of others, that might be otherwise overlooked. These beliefs contribute to mentors feeling “invaluable”;

“*Unless you’ve experienced it, you miss sometimes, the subtle clues that people send that they’re really not coping very well. So, I think the mentors are invaluable, in that we understand what it feels like. So, we can sort of come alongside them and say, ‘do you know what? It’s fine. It’s ok. We were where you are, this will pass. But it is not very pleasant when you’re going through it. Um… and that helps them, I think.*” (M1)

*Meaning and purpose sub-theme*

Mentors note that they have gained a sense of meaning and purpose from the group;

*“it’s this that’s given me a sense of achievement, accomplishment, purpose, my relationships” (M2).*

Mentors remarked that their purpose is to make others feel safe and valued;

*“It’s about making them feel safe and valued” (M1).*

Participants express gratitude to mentors, which enhances mentors’ beliefs that they have contributed to positive change;

“*the people who are taking the course, you can see the change taking place over a period of time. They’re not the same leaving the course as they are at the beginning of the course. And when they come and throw their arms around you and say thank you, you’ve made such a difference. I mean that is just brilliant, you know. Something good has come out of a bad situation and you can’t put a price on that really. Um… and sometimes you can’t…that doesn’t come through the data, you know…um yeah…they feel different and they can’t always say why*” (M4)

The influential change that mentors help to facilitate is impactful. One individual explained that she was feeling suicidal, however the course had reduced her depressive symptomology and suicidality, attributing the group to saving her life. Mentors believe themselves to be influential in this process of change, therefore creating a sense of purpose.

“*We’ve heard somebody’s story recently… they said that this time last year they were suicidal. They had written their letters to their family, that they were gonna go and that person, about half way through the course, they said something just switched with them and it just saved their life.*” (M2)

*Sense of transformation sub-theme*

Participants report having experienced a transformation and while they have not completely regained their abilities that were lost with an ABI, they declare a marked improvement;

“*yes, there are issues and yes, there are things that I can’t do, but I’m progressing beyond what I thought I could do because I’m finding other routes. So yeah, I think that that made me realise how far I’ve come in 5 years actually*.” (P7)

Participants note that the intervention facilitated new perspectives on happiness;

*“I went in thinking that can you really learn happiness, and I learnt about positivity … and to walk away as a happier person, I mean you can’t give a medicine for that.” (P8)*

and that the course had been life-changing;

*“My life is totally different.” (P6); “I know for a lot of people it changed their lives, I think it was pretty amazing, I think it taught everybody a lot” (P8).*

While participants recognise that they will experience failures in their attempts to perform new tasks, they describe a sense of acceptance and focus on the gains relating to failure, such as opportunity for growth;

“*I find that the more I stretch myself, the better I am. The more improvement that I make… That I am stretching myself with things I feel uncomfortable with. I say yes, and I make myself do it. Although I am stressed, I am nervous, I know its good for me. And so, I know I’m still making progress.”* (M2)

**Social Opportunity Theme**

*Relatedness*

Participants explain that having a shared understanding of ABI facilitates a comfortable, judgement-free and acceptance-based atmosphere, which in turn promotes of sense of unity based on equality;

*“I think if you want to express something you can talk to somebody here and they can understand it. And they don’t judge you…” (P1); “Everyone comes in at the same level.” (P3).*

This led to individuals conversing more and exploring their experiences of brain injury with others;

*“the difference it makes coming to a group like this and actually speaking to people who know what you’re on about and understand everything, and getting you’re voice.” (P4).*

Participants also found it helpful to meet others who had experienced ABI to get a sense of how they had rebuilt their lives following diagnosis;

*“it’s that meeting like-minded people and seeing how they have rebuilt their lives is a great help to me” (P6).*

Positive changes made by group members in turn provided hope of positive change for others. Mentors were viewed as inspirational, as participants felt that they had made significant progress in their recovery;

*“Yeah it’s exactly the same with the mentors as well. You can see them and say ‘right okay the level of improvement they have made and [see what] we can get to” (P4).*

*Understanding the self*

Participants described that group membership allowed them to explore their individual symptomology. Participants were able to recognise relevant symptoms – “*It was hearing what everyone was saying. It was allowing me to establish what my symptoms are*” (P4) – and explore confusing and difficult behaviour in a comfortable social arena;

*“I couldn’t explain myself before, whereas now I have outbursts of where I have got no breaks of… before I would say things and I would get so upset that I wouldn’t get over it, why have I done that? I didn’t understand why I was doing it. And I sort of isolated myself then. Sort of punishing myself. Whereas now I think it’s okay to have that blip.” (P2)*

*Helping others sub-theme*

Mentors were given the opportunity to help others, which as previously mentioned facilitated a sense of purpose and meaning. Though related to empowerment, helping others also relates to social opportunity as deriving meaning and purpose from helping others serves as a rationale to continue supporting others. In turn, this affords opportunity for relatedness and understanding of the self.

**Coping Theme**

*Reframing sub-theme*

Coping strategies learned during the intervention were utilized by both mentors and participants. Strategies such as grounding techniques, gratitude techniques, and humour were used to reframe thoughts, emphasising the facilitation and maintenance of positive emotion over negative emotion;

“*I am a lot happier, and because I have gone back to basics and hearing other people’s stories at how they appreciate just the simple things you know, it’s not raining outside, I’m really pleased its dry today, I’m putting a positive slant on things rather than a negative slant and I’ve been trying to do that a lot more so that was really useful for me.*” (P8)

While participants recognised that they would continue to experience negative emotion, they used these techniques in conjunction with acceptance-based strategies such as mindfulness to cope with negative aspects of one’s life;

*“negative things happen in everybody’s life, this is just a way a coping with it. So…that’s for me anyway. I have started to see the positives more than the negatives” (M3).*

Acceptance over one’s life circumstances was fostered using social support; “*We’re too harsh on ourselves…its quite good that we do meet up, because we’re able to remind each other*” (M2), changes in perspective; “*my depression had started to kick back in and after doing the course. [PARTICIPANT] said there are people there that are not as far along in the journey as yourself. So it puts things into perspective*” (M3) and humour;

*P5: “We can laugh. And because we can laugh because everyone is in the same boat, we are laughing at ourselves instead of laughing at someone.”*

*P1: “You’re not pushing yourself down-“*

*P5: “No”*

*P1: “Because your bringing yourself up out of that.”*

Participants found that the combination of acceptance and cultivation of positive emotion lead to the ability to overcome difficult circumstances;

*“I wouldn’t have got through the months I went through without this group. Without a shadow of a doubt.” (P2),*

as well as an overall increase in happiness and emotional regulation;

*“definitely in terms of keeping yourself happy when you are really depressed, you need to be able to fall back on positivity and how you pick yourself up from things, so I think the happiness group was amazing in that. I think it was also amazing in the way that, not that it just taught you happiness but you could go away and practice it in-between, so I thought that was really useful.” (Individual, P8)*

The ability to reframe thoughts led to individuals viewing anxious situations as a marker for challenge;

*“everybody has to fight their own battle, but you can get there and I think you can be happy no matter what.” (P8);*

*“If I have to go into a strange supermarket say in LOCATION or somewhere I haven’t been before then I get all of those feelings come back, umm but I can go into shops around here that I know on my own, unsupervised, and survive…And actually enjoy the experience” (P6)*

When participants found challenges difficult and overwhelming, they sought the support of others to aid coping;

“I felt so totally out of my depth in the beginning and yet not wanting anybody to see or to know I was out of my depth because I thought everybody’s coping really well….I felt I belonged, I felt I had the support to be myself and say whatever I felt, what I wanted to say, to join in the amount I wanted to join in and not be pressured into doing anything” (P6)

*Mobilisation of character strengths sub-theme*

Coping mechanisms such as reframing were underpinned by mobilisation of character strengths (for mentors). The shift in attention from negative to positive emotion was reinforced by recognising strength, instead of weakness;

“think when you’ve had an illness, and like us with the brain damage, afterwards everything is negative. Because you’re focusing on what you used to do and you can’t do it anymore. But then you read your strengths and you think ‘oh hang on a minute, you know” (M2)

In focusing attention on character strengths, participants can reframe their thoughts in terms of their abilities, not disabilities. Acceptance-based coping strategies were also underpinned by the mobilisation of character strengths. In identifying character strengths, participants can accept areas of weakness, which was not the case before participating in the group;

“*Those are my strengths. So…if I’m tired and I can’t get my words out one day, that’s okay. It wasn’t before, but it is now. So…yeah. And that helps. I think that really helps and when you can share your story with other people, they begin to share their story with you. And once it’s out there and they’ve spoken it, it’s not as scary anymore*” (M1)

**Cultivation of Positive Emotion Theme**

Positive emotions were cultivated when experiencing empowerment, social opportunity, coping and consolidation of skills. For example, achieving the ability to execute tasks was associated with happiness and joy;

*“I sat in the car and had this huge great big grin on my face…I didn’t want to go I just felt so so amazing, so happy, I literally I felt so happy because to me I had achieved something” (P2).*

The creation of a new social network and social participation also lead to an increased sense of happiness;

*“the friendships you make, and to walk away as a happier person (P8).*

Helping others was associated with positive feelings;

*“it’s helped me put it into perspective and it sounds awful but that made me feel good” (M3).*

Positive emotions were used as a coping mechanism when dealing with difficult situations;

*“sometimes I may laugh at something I done whereas before I would cry.” (P2).*

Mentorship was described as being “*a privilege*” (M2); it “*felt good*” to consolidate skills. Positive emotions also served as a reinforcer across each of these aspects. For example, enjoyment was associated with achievement, which was attributed to wanting to take on more challenges and activities;

“*it’s learning new things after an injury as well and you take on board- from that I enjoyed that, and ‘oh yeah you know, let’s do this’. It just seems to be, I don’t know whether it’s because I feel better in myself. It just seems to be getting better and better*” (P2)

**Consolidation of Skills Theme**

Mentors noted that reframing is not an automatic process, but one that requires careful deliberative action;

“*You forget to do it. It almost like.. it isn’t automatic. But then of course we did the august/September course and I’ve attended all of those. It reminds us of what we’re supposed to do. So even though the treatment is for other patients, we’re getting treatment at the same time. And it’s helping us to put things back into perspective as well.*” (M1)

Thus, mentoring allowed individuals to reinforce knowledge, serving as a reminder to maintain skills;

“*I’d started to only see the negatives again. Whereas this has helped me to see the positives as well as the negatives and then deal with the negatives then, isn’t it…It’s like layers, like I’m building upon layers and then I think oh yeah, I remember now. I almost needed it [positive psychotherapy] to consolidate the information myself.*” (M2)

Revisiting the course several times over through mentoring also allowed mentors to absorb new information that they believed they had missed during prior courses;

“*You just get little nuggets of information. Like somebody will say, just as a throwaway line, and you’ll think- what? And you say, sorry can you repeat that? And you think- Ah! I forgot all about that! Why aren’t I doing that? Um so I just get as much from it, well, probably more.*” (M4)

This process of reinforcement and consolidation strengthened skills learned during each course. Mentors believed that they were experiencing personal development because of this process;

*“Yeah, helps us to get stronger I think, doesn’t it. Cause each time we do it, we do get a little bit stronger.” (P3).*

**Barriers to Efficacy Theme**

*Stage in recovery sub-theme*

One participant (P7) noted that her stage in recovery impacted on how beneficial she found the group. She commented;

“*I think if I’d have had this 5 years ago, 4 years ago, even 2 years ago when I was at rock bottom. I think it would have been such a great help…*” (P7)

P7 stated that her ABI was misdiagnosed and so did not receive any clinical intervention for several years following her injury. Consequently, her recovery was self-managed.

“*I could have really done with it back then but obviously I had no access to it, because they didn’t realise that I had a brain injury, so I think that, I did all my, sort of, stuff myself.*” (P7)

As a result, P7 believed that she was further along in her recovery compared to other group members. For instance, when others were exploring their emotions and issues with their ABI experience in a group forum, P7 became frustrated with displays of negative emotions such as crying, and felt this had a negative consequence on her own emotional state;

*“each time I was coming out of there more negative, because she’d spent so much time talking about how she was down”.*

P7 continues that she had played an active role in her recovery by consciously choosing to motivate herself towards progress, and P7 felt frustrated when others displayed negative emotions;

“*you know, well, I was like that 4 years ago and its not gonna get better like that… I sort of came to a road and I knew I could either go this way and let it sort of spiral out of control or, if I turn left I could actually make something out of myself. And obviously I had to turn left because I knew all of the damage it could do to my family and to my partner and all of that*.”

This session was early on in the intervention and P7 noted that she had not engaged with subsequent activities as result of the outcome of this particular session. P7 indicated that efficacy of the intervention might be contingent upon a stage in recovery;

*“I didn’t go to the mindfulness one. Um, because I really love mindfulness and … I didn’t want [to witness others’ negative emotions during] mindfulness, because I enjoy it too much*.

However, P7 explained that although she felt further along in recovery and part of the course was less relevant to her, she still found it helpful to compare her own progress with others. In that sense, she felt grateful for her own progress;

*“I feel further along with it. And I felt that even though it was really good for me to see that actually I’m not as bad as I think, other people.”*

*Location and accessibility sub-theme*

Many participants noted that the location of the intervention was difficult to access. The course was not located in proximal locations for some, which meant travelling long distances to participate in the course. Whilst participants do not mention that this led to drop-outs, it does suggest that accessibility is difficult for participants with mobility and transportation issues. Participants further noted that parking facilities at the location were extremely limited. Even with disabled badges, parking was still considered to be an issue; “*she had a disability badge and she still wouldn’t be able to park so well… parking was a major thing there*.” (P7).
